# Metabolic diversity and adaptation of carbon-fixing microorganisms in extreme glacial cryoconite

**DOI:** 10.1093/ismeco/ycaf056

**Published:** 2024-03-30

**Authors:** Yuying Chen, Yongqin Liu, Mukan Ji, Zhihao Zhang, Tingting Xing, Hongan Pan, Keshao Liu, Yueang Li, Pengfei Liu

**Affiliations:** Center for the Pan-Third Pole Environment, Lanzhou University, Lanzhou 730000, China; Center for the Pan-Third Pole Environment, Lanzhou University, Lanzhou 730000, China; State Key Laboratory of Tibetan Plateau Earth System, Environment and Resources, Institute of Tibetan Plateau Research, Chinese Academy of Sciences, Beijing 100101, China; College of Resources and Environment, University of Chinese Academy of Sciences, Beijing 100049, China; Center for the Pan-Third Pole Environment, Lanzhou University, Lanzhou 730000, China; State Key Laboratory of Tibetan Plateau Earth System, Environment and Resources, Institute of Tibetan Plateau Research, Chinese Academy of Sciences, Beijing 100101, China; State Key Laboratory of Tibetan Plateau Earth System, Environment and Resources, Institute of Tibetan Plateau Research, Chinese Academy of Sciences, Beijing 100101, China; Laboratory of Soil Microbial Geography, School of Geographical Sciences, Nanjing Normal University, Nanjing 210023, China; State Key Laboratory of Tibetan Plateau Earth System, Environment and Resources, Institute of Tibetan Plateau Research, Chinese Academy of Sciences, Beijing 100101, China; Faculty of Forestry, Natural Resource Conservation, University of British Columbia, 2300 West Mall, Vancouver BC V6T 1Z4, Canada; Center for the Pan-Third Pole Environment, Lanzhou University, Lanzhou 730000, China

**Keywords:** Tibetan plateau, cryoconite, microbial community, carbon fixation, energy sources, DNA-SIP

## Abstract

Understanding the diversity and functionality of carbon-fixing microorganisms in glacial ecosystems is crucial for elucidating carbon cycling processes in extreme environments. This study investigates the composition, diversity, and metabolic potential of carbon-fixing microorganisms in Tibetan cryoconite. Through metagenomic sequencing, we identified 13 carbon-fixing metagenome-assembled genomes spanning ten known and three unclassified genera. Deoxyribonucleic acid -stable isotope probing experiments with ^13^C-labeled sodium bicarbonate confirmed the metabolic activity of key genera, including Cyanobacteria (*Microcoleus* and *Phormidesmis*) and Proteobacteria (*Rhizobacter* and *Rhodoferax*). Our results reveal a diverse array of carbon fixation pathways, with the Calvin–Benson–Bassham cycle and 3-hydroxypropionate bicycle being the most prominent. In addition to photoautotrophic microorganisms, chemoautotrophic microorganisms also contribute to carbon fixation through mechanisms such as sulfur oxidation and atmospheric reducing gas utilization. The study highlights the adaptability of microbial communities to varying environmental conditions, including fluctuations in oxygen, light, and substrate availability. The findings underscore the complex interplay between carbon fixation pathways and environmental factors in cryoconite ecosystems. It also emphasizes the importance of exploring alternative carbon fixation pathways to gain a more comprehensive understanding of carbon cycling in these harsh and dynamic ecosystems.

## Introduction

Cold environments, particularly glaciers, host crucial microbial processes that drive carbon fixation, significantly influencing global carbon cycling [1]. Glaciers, storing over 10^4^ Pg of organic carbon globally, have seen a dramatic increase in carbon release due to global warming [1]. This release has profound implications for downstream ecosystems, tipping net community metabolism towards heterotrophy as organic carbon inputs fuel increased respiration [2, 3]. On glacier surfaces, microbial communities in cryoconite—granular sediment comprising both mineral and biological material—are central to the conversion of inorganic carbon into organic carbon [4]. Cryoconite represents the most metabolically active environment on glaciers, where carbon fixation is primarily carried out by autotrophic microorganisms [5, 6]. These microbes, including photoautotrophs that harness light energy and chemoautotrophs that derive energy from chemical reactions, are integral to sustaining organic carbon levels in these ecosystems [7].

Cryoconite ecosystems across polar and alpine regions harbor diverse carbon-fixing microorganisms [8]. Cyanobacteria, the dominant photoautotrophs in these environments, perform oxygenic photosynthesis and constitute a significant portion of cryoconite bacterial communities [8, 9]. Their activity has been validated through ribosomal ribonucleic acid (rRNA) analyses [10, 11]. Notably, the taxonomic composition of Cyanobacteria varies geographically. In Asian glaciers, cryoconite hosts multiple cyanobacterial lineages, including *Leptolyngbyaceae*, *Chamaesiphonaceae*, *Microcoleaceae*, and *Pseudanabaenaceae*, whereas polar glaciers are primarily dominated by *Leptolyngbyaceae* [12]. Beyond Cyanobacteria, other photosynthetic microorganisms, particularly from the phylum Proteobacteria, contribute to carbon fixation in cryoconite. These microbial groups exhibit significant geographical specificity, with certain taxa, such as *Rhizobiales*, more abundant in specific locations like the Athabasca Glacier in Canada compared to Andean glaciers in Chile [13, 14]. Chemoautotrophs, which oxidize compounds like ammonia, hydrogen, and sulfur, further diversify the carbon-fixing community, with their activity confirmed by transcriptomic data [10, 15, 16]. The presence of these varied microbial groups indicates that distinct environmental conditions within cryoconite shape specialized carbon-fixing communities.

Despite the identification of numerous carbon-fixing microorganisms in cryoconite, the functional dynamics of these communities remain underexplored. Deoxyribonucleic acid stable-isotope probing (DNA-SIP) provides a powerful tool to investigate the active microorganisms involved in carbon fixation by tracking labeled carbon substrates into DNA [17, 18]. While DNA-SIP has successfully identified active carbon-fixing bacteria in various ecosystems [19, 20], its application in cryoconite ecosystems is still in its infancy.

Microorganisms utilize a variety of carbon fixation pathways, with eight common pathways documented, including the CBB cycle, reductive citric acid (rTCA) cycle, and Wood–Ljungdahl (WL) pathway, 3-hydroxypropionate (3-HP) bicycle, 3-hydroxypropionate/4-hydroxybutyrate (3-HP/4-HB) cycle, dicarboxylate/hydroxybutyrate (DC/4-HB) cycle, reductive glycine (rGly) pathway, and reversed oxidative tricarboxylic acid (roTCA) cycle [21, 22]. The CBB cycle, primarily used by oxygenic photoautotrophs, is the most well-characterized pathway in cryoconite ecosystems [23, 24]. Other pathways, such as the rTCA cycle and WL pathway, are employed by chemoautotrophs and vary across niches due to differences in energy demands and oxygen sensitivity [7, 16]. Despite growing knowledge of these pathways, studies on their presence and activity in cryoconite on the Tibetan Plateau remain limited.

The Tibetan Plateau, holds the largest glacier area outside the polar regions, spanning approximately 100 000 km^2^ [25]. This region is undergoing rapid glacial retreat, with 80% of its glaciers in decline [26]. This region presents dynamic environmental challenges for microbial communities due to significant diurnal and seasonal climate variations. We hypothesize that cryoconite on the Tibetan Plateau harbors a diverse array of carbon-fixing microorganisms that utilize multiple energy sources and carbon fixation pathways to adapt to these extreme environmental conditions. To test this hypothesis, we conducted a comprehensive study, collecting cryoconite samples from various locations across the Tibetan Plateau to investigate the diversity, energy sources, and carbon fixation pathways of these microorganisms.

## Materials and methods

### Sampling, deoxyribonucleic acid extraction, and metagenomic sequencing

Fifteen cryoconite samples were collected from Palong glacier (hereafter abbreviated as PL), Qiangyong glacier (QY), Dongkemadi glacier (DKMD), Longxiazailongba glacier (LXZ), and Dunde glacier (DD) on the Tibetan Plateau during the summers of 2016 to 2020 (Supplementary Fig. S1 and Table S1). These five glaciers span from 28.87°N to 38.11°N and range from 4805 meters to 5379 meters above sea level, providing a comprehensive representation of spatial characteristics in the Tibetan Plateau.

Three cryoconite holes were randomly selected as biological replicates and collected using a stainless steel scoop and placed in pre-cleaned 1-L polycarbonate bottles (Nalgene). Total DNA for cryoconite samples was extracted and sequenced. The details of sample collection, DNA extraction, and sequencing are provided in the Supplementary method**.**

### Metagenomic data analysis

The quality control process was performed using the software Trimmomatic (v0.39) with the parameters (LEADING:3 TRAILING:3 SLIDINGWINDOW:5:20 MINLEN:50) [27]. After quality control, MEGAHIT (v1.2.9, https://github.com/voutcn/megahit) was used for de novo assembly of the high-quality sequences with K-mer settings as follows: k-min 35, k-max 95, k-step 20 [28]. The software Prodigal (v2.6.3) was used to predict open reading frames (ORFs) in contigs that were longer than 500 bp [29]. The non-redundant gene catalog was constructed using MMseqs2 software (version 13.45111) as follows: all predicted ORFs were combined, and dereplicated by clustering at 80% aligned region with 95% nucleotide identity with the parameters “-e 0.001 --min-seq-id 0.9 -c 0.80” [30]. The longest sequence in each cluster was selected as the representative sequence (Unigene) to form the non-redundant gene catalog for all samples [31]. The abundances of these genes across 15 metagenomes were measured by transcripts per million (TPM) calculated by Salmon software (v1.9.0) in metagenome mode [32]. Briefly, reads mapped to genes were normalized by gene length and total mapped number following the formula below:


$$ {TPM}_i=\frac{r_i/{l}_i}{\sum_j\left({r}_j/{l}_j\right)}\times{10}^6 $$

in which *r_i_* refers to the reads mapped to gene *i, l_i_* is the length of gene *i*, and ${\sum}_j\left({r}_j/{l}_j\right)$ corresponds to the sum of mapped reads to genes normalized by gene length.

Metagenomic assembled contigs, with a minimum length of 500 bp, maximum length of 272 452 bp and averaging length of 1249 bp, were binned using MetaWRAP v1.3.2 with the most effective binning methods, MetaBAT 2 (v2.12.1) [33]. A total of 1890 metagenome-assembled genomes (MAGs) were obtained. The completeness and contamination of MAGs were assessed using CheckM (v1.1.3) [34]. Only high-quality MAGs with completeness greater than 80% and contamination less than 10% were considered in this study. The MAGs were then dereplicated using the dRep software (v3.2.0), resulting in a total of 157 MAGs [35]. The Prokka software was utilized for ORF calling and gene prediction [36]. We used the Burrows–Wheeler aligner (BWA, v0.7.17) [37] to map the reads from each sample against all contigs. The number of reads mapped to each contig was calculated using SAMtools (v0.1.19) [38]. The abundance of the MAG was calculated by summing the number of reads that mapped to contigs belonging to that genome. The proportion of reads mapped to MAGs was calculated by summing the abundance of all MAGs and then dividing by the total number of reads in the metagenome. The relative abundance of each MAG in each sample was determined as the percentage of reads recruited by a MAG relative to the total reads recruited by all MAGs in that sample.

### Functional annotation of metagenome-assembled genomes and non-redundant genes

The MAGs were annotated using the Kyoto Encyclopedia of Genes and Genomes (KEGG) Orthologies (KOs) database (release 107.1) via the “annotate” function in EnrichM v0.5.0 [39]. Module completeness was calculated using EnrichM’s “classify” function, which evaluates the integrity of KEGG modules—sets of genes organized by steps in metabolic pathways as defined by KEGG. For carbon fixation, considering the potential incompleteness of MAGs, the module completeness was required to be ≥50% along with the presence of the pathway’s marker gene. For ATP synthesis, nitrogen metabolism, and sulfur metabolism, complete completeness (100%) was mandated to avoid overestimating metabolic capacities. For photosynthesis and CO oxidation, the marker genes were screened. The annotation of carbohydrate-active enzymes (CAZymes) in MAGs was performed using the dbCAN3 database (https://bcb.unl.edu/dbCAN2) with HMMER v3.3.1 [40].

To obtain a more comprehensive understanding of carbon fixation pathways, non-redundant genes were also annotated against the KOs database using Kofamscan (v1.3.0, https://github.com/takaram/kofam_scan). Only marker genes for each carbon fixation pathway were considered to avoid overestimation of pathway potential due to gene overlap between genes in carbon fixation pathways and organic carbon decomposition pathways. Detailed information on the marker genes and module ID is provided in Supplementary Table S2.

### Phylogenetic analyses

The taxonomy of the 157 MAGs was classified using the GTDB-tk tool (v2.1.1) aligned to the GTDB database (version r207) [41]. Phylogenetic relationships among the MAGs were inferred by constructing a maximum-likelihood tree based on 120 bacterial and 122 archaeal marker genes identified by GTDB-tk. Concatenated multiple sequence alignments were performed using the GTDB-Tk align module. The phylogenetic tree was constructed using FastTree v2.1.11 with default parameters [42] and visualized using the Interactive Tree of Life (iTOL) webtool [43].

Additionally, a phylogenetic tree was constructed to identify the forms of ribulose-1,5-bisphosphate carboxylase/oxygenase (RuBisCO), the key enzyme in the CBB cycle [44]. Protein sequences retrieved from MAGs were aligned with reference sequences using Muscle v3.8.31 [45]. The phylogenetic tree was constructed using the maximum-likelihood method in FastTree v2.1.11 [42] and visualized using iTOL webtool [43]. The protein sequences of RuBisCO from the MAGs, along with the reference sequences from the work of Bay et al. [46], are provided in Supplementary Table S3.

### Microcosm experiments

To identify active carbon-fixation microorganisms within the complex cryoconite microbial community, the DNA-SIP method was applied to cryoconite samples. Briefly, 40 g of fresh cryoconite with 80 ml sterilized ultrapure water were added to 125 ml serum bottles. The bottles were sealed with sterilized parafilm to allow gas exchange. For sacrificial sampling, 12 bottles were incubated with NaH^13^CO₃ (99% ^13^C-content, Sigma-Aldrich, Darmstadt, Germany) at a final concentration of 2.5 mM, with 3 bottles serving as biological replicates for each time point. As controls, 4 bottles were incubated with unlabeled NaH^12^CO₃ at the same concentrations, with 1 bottle per time point. All incubations were performed at 15°C under an illumination intensity of 2000–2500 lx. The bottles were destructively sampled on the 0^th^, 20^th^, 40^th,^ and 60^th^ days. After incubation, all subsamples were stored at −20°C for molecular analyses. Detailed description of carbon isotopic composition (δ^13^C) measure, DNA extraction, DNA-SIP fractionation, Quantitative Polymerase Chain Reaction (qPCR), PCR amplification, and high-throughput sequencing and data analysis methods are provided in Supplementary methods.

### Statistical analysis

The significance test between carbon fixation pathway marker genes was performed by one-way analysis of variance (ANOVA) with Tukey’s post hoc test. All statistical analyses were performed using R version 4.3.2.

## Result

### Potential carbon-fixing metagenome-assembled genomes in Tibetan cryoconites

**Figure 1:**
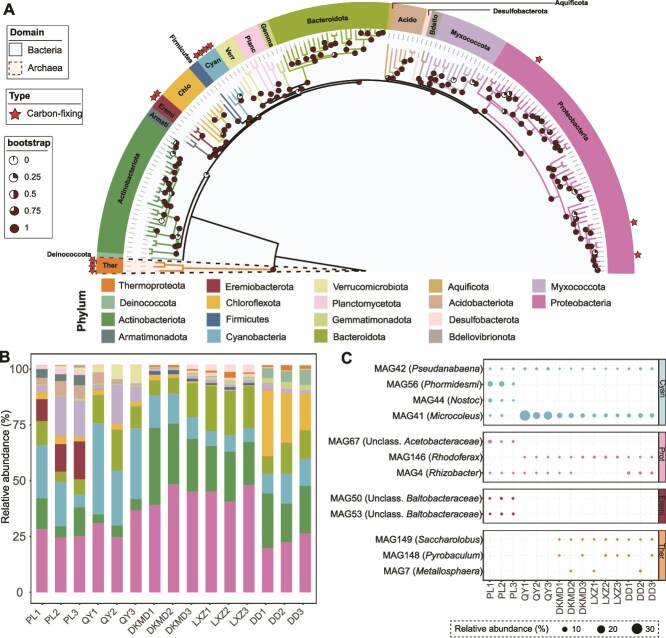
Phylogenetic tree of MAGs and potential carbon-fixing MAGs. (A) Phylogenetic tree generated from 157 MAGs. The clade background color represents the domain of each MAG. Color strip in the outer ring and branch colors represents the phylum of each MAG. Potential carbon-fixing MAGs are marked with stars. Bootstrap values, based on 1000 iterations, are displayed by pie charts. (B) Relative abundance of microbial taxa at the phylum level across 15 samples. (C) Bubble plot displaying the relative abundance of potential carbon-fixing MAGs in the 15 samples. Phylum abbreviations: Cyanobacteria (Cyan), Proteobacteria (Prot), Eremiobacterota (Eremi), and Thermoproteota (Ther). Abbreviations of sample locations: Palong glacier (PL), Qiangyong glacier (QY), Dongkemadi glacier (DKMD), Longxiazailongba glacier (LXZ), and Dunde glacier (DD).

From 15 metagenomic samples, 157 high-quality MAGs were obtained, with an average coverage of 4.6% (Supplementary Table S4). These MAGs spanned 13 known bacterial phylum and 1 archaeal phylum (Thermoproteota) (Fig. 1A; Supplementary Table S4). Predominant phyla included Proteobacteria (51 MAGs, average 33.0% of high-quality MAGs), Actinobacteriota (27 MAGs, 16.8%), Cyanobacteria (4 MAGs, 15.6%), and Bacteroidota (23 MAGs, 12.1%) (Fig. 1B).

Among these, 16 MAGs contained the RuBisCO marker genes associated with carbon fixation pathways, and the completeness of the carbon fixation pathway was greater than 50% (Supplementary Tables S5 and S6). Four MAGs encoded type III RuBisCO, which is not involved in carbon fixation (Supplementary Fig. S2), leaving 12 MAGs as potential carbon-fixing candidates (Fig. 1C). These potential carbon fixation MAGs contributed between 8.6% and 40.4% in each sample, with the highest contribution observed in the QY glacier and the lowest in the LXZ glacier (Supplementary Fig. S3).

Specifically, MAG41 (*Microcoleus*) exhibited the highest relative abundance, averaging 11.56% across communities and reaching 28.80% in QY glacier (Fig. 1C). MAG56 (*Phormidesmis*) and MAG42 (*Pseudanabaena*) followed, with average relative abundances of 2.05% and 1.35%, respectively. MAG56 showed its highest relative abundance in PL glacier (10.2%), while MAG42 was most abundant in QY glacier (2.9%). MAG4 (*Rhizobacter*) had a relative abundance of 0.80% and was the most abundant in DD glacier (3.4%). Additional MAGs, including MAG44 (*Nostoc*), MAG67 (unclassified *Acetobacteraceae*), MAG50 (Unclassified *Baltobacteraceae*), and MAG53 (unclassified *Baltobacteraceae*), exhibited average relative abundances of 0.66%, 0.65%, 0.32%, and 0.32%, respectively. MAG146 (*Rhodoferax*), with an average relative abundance of 0.69%, showed the highest abundance in LXZ glacier (2.50%). Archaeal MAGs from the phylum Thermoproteota were identified but with very low relative abundances, including MAG7 (*Metallosphaera*, average relative abundances of 0.26%), MAG148 (*Pyrobaculum*, 0.26%), and MAG149 (*Saccharolobus*, 0.19%; Fig. 1C).

### Active carbon fixation microorganisms in deoxyribonucleic acid stable-isotope probing

**Figure 2:**
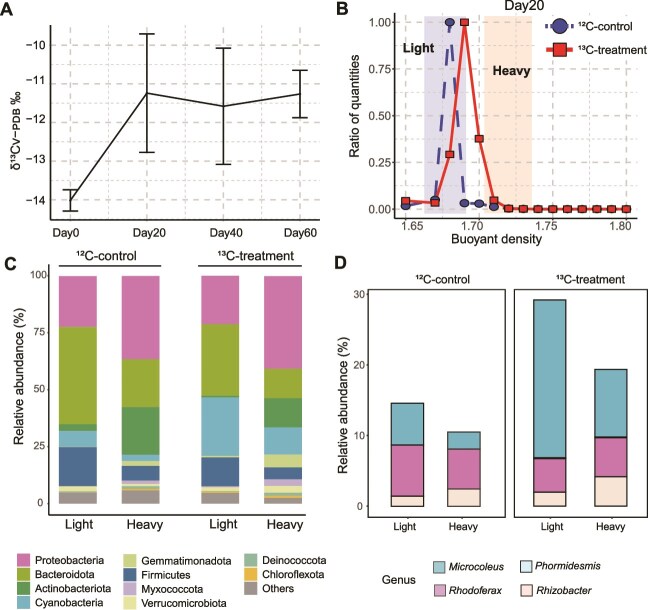
Microcosm experiments combining DNA-SIP to explore active carbon-fixing microorganisms. (A) Variation of δ^13^OC over the 60-day incubation period. Error bars are standard deviations from three replicates. (B) Increased DNA buoyant density of normalized 16S rRNA genes of ^12^C-contol and ^13^C-treatment at Day20. The solid lines represent the ^13^C-treatment and the dashed lines represent the ^12^C-controls. The shaded area represents light and heavy fractions. (C) Relative abundance of microbial taxa of ^12^C-control and ^13^C-treatment from different density fractions at phylum level. (D) The relative abundances of genera identified as MAGs in the metagenome were compared between the heavy and light fractions in both the ^12^C-control and the ^13^C-treatment.

Over the 20-day incubation, the δ^13^C of organic carbon increased from −14‰ to −11‰ and then stabilized, indicating active carbon fixation (Fig. 2A). Uptake of ^13^C-labeled bicarbonate was observed during incubation, resulting in an increased DNA buoyant density in the ^13^C treatment (1.6914 g ml^−1^) compared to the ^12^C control (1.6820 g ml^−1^) (Fig. 2B). Three ^12^C-light fractions (density 1.6726 g ml^−1^, 1.6820 g ml^−1^, and 1.6914 g ml^−1^, respectively) in the ^12^C-control represent the unlabeled community. In the ^13^C-experiment, three ^13^C-heavy fractions (density 1.7007 g ml^−1^, 1.7077 g ml^−1^, and 1.7285 g ml^−1^, respectively) represent the labeled community. The ^12^C-heavy fraction (density 1.7077 g ml^−1^) acts as a key comparison with the ^13^C-heavy fractions to confirm that the microbial communities identified in the ^13^C-heavy fractions result from the uptake of the labeled isotope rather than the influence of high G + C content. Bacteria affiliated with Proteobacteria, Bacteroidota, Actinobacteriota and Cyanobacteria dominated the ^13^C-heavy fractions (averaged relative abundance of 40.7%, 13.0%, 12.8%, and 12.0%, respectively; Fig. 2C). Bacteroidota, Proteobacteria, Firmicutes, and Cyanobacteria dominated the ^12^C-light fractions (42.8%, 22.3%, 17.2%, and 6.9%, respectively). The ^12^C-heavy fractions were dominated by Proteobacteria, Bacteroidota, Actinobacteriota, and Firmicutes, with averaged relative abundance of 36.6%, 21.0%, 21.0%, and 6.5%, respectively. Proteobacteria, Cyanobacteria, Gemmatimonadota, Myxococcota, and Verrucomicrobiota showed higher abundance in the ^13^C-heavy fractions compared to the ^12^C-heavy fractions (Fig. 2C).

At the genus level, *Microcoleus*, *Polaromonas*, *Rhodoferax*, and *Gemmatimonas* dominated the ^13^C-heavy fractions with an averaged relative abundance of 9.9%, 5.9%, 5.7%, and 5.6%, respectively (Supplemental Fig. S4 and Table S7). *BSV13*, *Flavobacterium*, *Rhodoferax*, and *Paludibacter* dominated the ^12^C-light fractions (16.3%, 11.4%, 7.1%, and 6.7%, respectively). The ^12^C-heavy fractions were dominated by *BSV13*, *Rhodoferax*, *Flavobacterium*, and *Rhizobacter* (10.1%, 5.5%, 3.4%, and 2.4%, respectively). By comparison, 34 genera with relative abundances above 0.1% in the ^13^C-heavy fractions showed higher relative abundances compared to the ^12^C-heavy fraction (Supplementary Table S8). Among them, four genera were also identified in the MAGs, including *Microcoleus*, *Rhodoferax*, *Rhizobacter*, and *Phormidesmis* (Fig. 2D).

### Energy-related metabolic potential of carbon-fixing metagenome-assembled genomes

**Figure 3:**
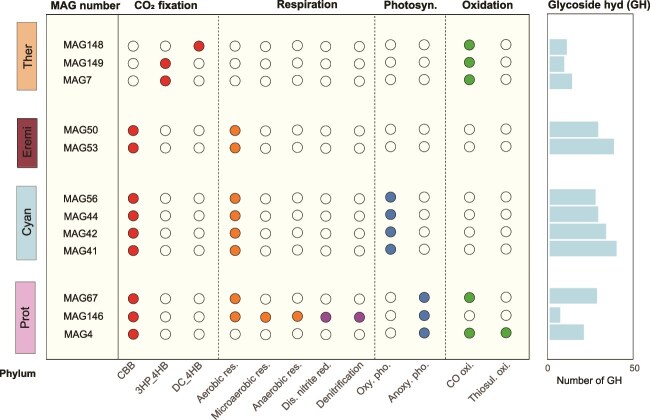
Metabolic strategies of 12 potential carbon fixation MAGs. The horizontal axis lists different metabolic processes, including CO_2_ fixation, respiration, photosynthesis (photosyn), oxidation of CO and sulfide. The vertical axis identifies each MAG. The different color bars on the left represent the taxonomic classification of each MAG. Solid circles indicate that the corresponding MAGs possess specific metabolic functions. Hollow circles indicate the absence of these functions in the corresponding MAGs. The metabolic processes are abbreviated as follows: Calvin–Benson–Bassham cycle (CBB), 3-hydroxypropionate/4-hydroxybutyrate cycle (3-HP/4-HB), dicarboxylate/hydroxybutyrate cycle (DC/4-HB), aerobic respiration (aerobic. res.), microaerobic respiration (microaerobic res.), anaerobic respiration (anaerobic res.), dissimilatory nitrate reduction (dis. nitrite red.), oxygenic photosynthesis (oxy. pho.), anoxygenic photosynthesis (anoxy. pho.), carbon monoxide oxidation (CO oxi.), thiosulfate oxidation (thiosul. oxi.). Phylum abbreviations are as follows: Proteobacteria (Prot), Cyanobacteria (Cyan), Eremiobacterota (Eremi), and Thermoproteota (Ther).

To explore the autotrophic strategies supporting life in cryoconite, we assessed the energy conservation potential of the identified carbon-fixing MAGs (Fig. 3 and Supplementary Tables S5 and S6). Aerobic respiration was evident in eight potential carbon-fixing MAGs, as indicated by the presence of genes encoding the complete cytochrome c oxidase (M00155). MAG146, affiliated with *Rhodoferax*, harbored genes supporting both microaerobic and anaerobic respiration, indicated by cytochrome c oxidase cbb3-type (M00156) and NADH:quinone oxidoreductase (M00144), respectively. This MAG also exhibited complete pathways for dissimilatory nitrate reduction (M00530) and denitrification (M00529). Oxygenic photosynthetic marker genes (*psaAB* and *psbABCD)* were identified in all four MAGs affiliated with Cyanobacteria (Fig. 3 and Supplementary Table S5). Genes associated withnanoxygenic photosynthesis (*pufLM*) were detected in three MAGs, including MAG67 (affiliated with unclassified *Acetobacteraceae*)*,* MAG146 (*Rhodoferax*)*,* and MAG4 (*Rhizobacter*). Five MAGs were demonstrated to be capable of carbon monoxide (CO) oxidation, as evidenced by the presence of aerobic CO dehydrogenase encoding genes (*coxLMS*). These include MAG148 (*Pyrobaculum*), MAG149 (*Saccharolobus*), MAG7 (*Metallosphaera*), MAG67 (unclassified *Acetobacteraceae*), and MAG4 (*Rhizobacter*). Genes required for chemolithotrophy were found in MAG4 (*Rhizobacter*), which contained a complete thiosulfate oxidation (SOX) system (M00595) (Fig. 3 and Supplementary Table S6).

All potential carbon-fixing MAGs encoded a diverse array of glycoside hydrolase (GH) genes, predicted to hydrolyze substrates such as beta-glucan, xylan, beta-mannan, peptidoglycan, exopolysaccharide (EPS), cellulose, chitin, xyloglucan, starch, glycogen, pectin, polyphenols, and sucrose (Fig. 3 and Supplementary Table S9).

### Carbon fixation pathways at metagenome-assembled genomes and metagenome level

The identified potential carbon-fixing MAGs were involved in three distinct carbon fixation pathways: the CBB cycle, the 3-HP/4-HB cycle, and the DC/4-HB cycle (Fig. 3). The CBB cycle was the most prevalent, represented in 9 MAGs, while the 3-HP/4-HB and DC/4-HB cycles were less common, present in 2 and 1 MAGs, respectively. The genetic potential for the CBB cycle was distributed across multiple bacterial phyla, including Proteobacteria (MAG4, MAG146, and MAG67), Cyanobacteria (MAG42, MAG44, MAG53, and MAG56), and Eremiobacterota (MAG50 and MAG53). The 3-HP/4-HB cycle was detected in MAG7 and MAG149, both affiliated with Thermoproteota, while the DC/4-HB cycle was identified in MAG148, also belonging to Thermoproteota (Fig. 3).

**Figure 4:**
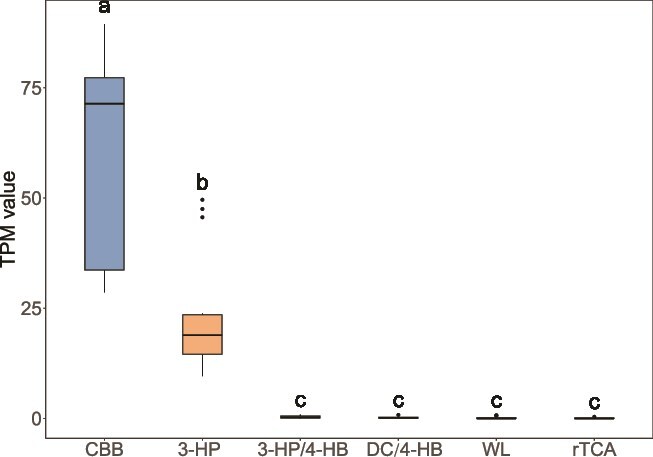
Comparison of TPM value of carbon fixation pathways at metagenomic level. Different carbon fixation pathways are distinguished by colors of the graph. The horizontal axis represents different carbon fixation pathways, while the vertical axis represents the TPM value. Different letters represent significant differences at *P* < .05 (one-way ANOVA with Tukey’s post hoc test).

Since the MAGs obtained through binning account for only a portion of the total reads (Supplementary Table S3), we conducted a search for marker genes involved in carbon fixation pathways using all contigs at the metagenome level to provide a more comprehensive understanding. Marker genes for six carbon fixation pathways were identified (Fig. 4). Among these, the CBB cycle and the 3-HP bicycle were the predominant pathways, with significantly higher TPM values compared to other pathways, averaging 59.5 and 23.1, respectively (*P* < .001, one-way ANOVA with Tukey’s post hoc test). The abundance of the CBB cycle was significantly higher in the PL, QY, and DD glaciers compared to the LXZ and DKMD glaciers, nearly double in TPM value (*P* < .001, one-way ANOVA with Tukey’s post hoc test; Supplementary Fig. S5). Similarly, the 3-HP bicycle exhibited significantly higher abundance in QY glacier, exceeding other glaciers by more than twofold (*P* < .001, one-way ANOVA with Tukey’s post hoc test). In contrast, other carbon fixation pathways were present at relatively low abundances, including the 3-HP/4-HB cycle (TPM value of 0.41), WL pathway (0.32), DC/4-HB cycle (0.27), and rTCA cycle (0.17) (Fig. 4).

## Discussion

### Diverse carbon-fixing microorganisms in the Tibetan cryoconite

In this study, we identified 12 carbon-fixing MAGs representing 9 known genera and 3 unclassified genera (Fig. 1C). The most abundant carbon-fixing microorganisms in cryoconite were affiliated with photosynthetic Cyanobacteria (Fig. 1C). This is consistent with global studies that identify Cyanobacteria as primary producers in cryoconite environments [12, 47, 48]. Specifically, MAG41, belonging to the genus *Microcoleus,* emerged as both ubiquitous and dominant in Tibetan cryoconite, consistent with previous findings identifying *Microcoleus* as a major group in Asian glaciers [12]. The inorganic carbon fixation activity of this genus was confirmed through ^13^C incubation (Supplementary Fig. S4).

Proteobacteria, particularly members of the genera *Rhizobacter* and *Rhodoferax*, also played significant roles in primary production (Fig. 1C). These genera have been detected in cryoconite from diverse cold regions, including the Arctic, Antarctic, and Tibetan Plateau [16, 49–51]. While carbon fixation by *Rhizobacter* in cryoconite has not been previously reported, our findings indicate that this genus harbors a complete CBB cycle pathway (Supplementary Table S5), and its carbon fixation activity was verified through ^13^C incubation (Supplementary Fig. S4). The presence of *Rhizobacter*, typically associated with rhizospheric environments, in Tibetan cryoconite may result from wind-borne deposition from glacier forelands [52]. Similarly, *Rhodoferax*, known as purple non-sulfur bacteria, demonstrated carbon fixation activity via ^13^C incubation (Supplementary Fig. S4). This genus has been reported to fix carbon in subglacial and proglacial environments [53].

Additional carbon-fixing potential was observed in members of Thermoproteota (MAG149, MAG148, and MAG7) and Eremiobacterota (MAG50 and MAG53) (Fig. 1C). Thermoproteota has been identified as a dominant archaeal phylum in cryoconite microbiomes of the Northern Hemisphere [51], while Eremiobacterota has been associated with chemolithoautotrophic carbon fixation in Antarctic soils [54]. Although the activity of these genera was not directly confirmed via DNA-SIP experiments, their carbon fixation potential under specific conditions remains plausible.

In addition, certain genera exhibiting ^13^C uptake were not captured in the MAGs (Supplementary Table S7). This likely reflects challenges associated with genome binning, as some reads may not assemble into MAGs [55]. The completeness and contamination of successfully binned MAGs may also affect the assessment of carbon fixation potential. For example, gaps in genome assembly or missing contigs might lead to underestimation of pathway completeness, while misassignments of unrelated genes could result in overestimation [56]. Future research could explore co-assembly strategies to further improve MAG completeness and recover a greater diversity of rare microbial populations. Furthermore, based on taxonomic classification, several genera not included in MAGs have previously been reported to be capable of carbon-fixing. For example, *Polaromonas* has been recognized for its carbon fixation capabilities in a previous study [57]. Additionally, cross-feeding during incubation cannot be ruled out, which may explain the presence of heterotrophic microorganisms in the ^13^C-heavy fraction. Thus, only focusing on the genera identified as having carbon fixation potential through MAGs may underestimate the diversity of carbon-fixing microorganisms in cryoconite. Future research should incorporate complementary methods to achieve a more comprehensive understanding of carbon-fixing microorganisms.

### Metabolically flexible mixotrophic microorganisms drive cryoconite carbon fixation

Cryoconite carbon-fixing microorganisms on the Tibetan Plateau utilize diverse energy sources, including light, chemical energy, trace atmospheric gas and carbohydrates, expanding our understanding of primary production in glacial cryoconite (Fig. 3). Photoautotrophic Cyanobacteria were identified as the primary carbon fixers, consistent with findings from Arctic and Alpine cryoconite [24]. Additionally, aerobic anoxygenic photosynthetic bacteria (AAPB), such as MAG146 (affiliated with *Rhodoferax*), were detected. Members of the genus *Rhodoferax* with anoxygenic photoautotrophic capabilities have been reported in Antarctic microbial mats [58]**.**

Genes for CO oxidation were identified in five MAGs belonging to Thermoproteota and Proteobacteria (Fig. 3). This finding aligns with reports of Thermoproteota engaging in CO oxidation in terrestrial ecosystems [59]. Such oxidation may generate reducing equivalents to support CO_2_ fixation or enhance microbial survival under nutrient-limited conditions [60, 61]. In glacial environments, CO can form rapidly through photochemical reactions of organic carbon in snowmelt, offering a selective advantage to microorganisms by providing an alternative energy source [62, 63].

Chemoautotrophic capacity for carbon fixation was limited, with only MAG146 (*Rhodoferax*) possessing a complete thiosulfate oxidation pathway (Fig. 3 and Supplementary Table S5). *Rhodoferax* has been shown to fix carbon using sulfides as electron donors in high-sulfur cryoconite [16]. Glacial weathering may release trace amounts of reduced sulfur compounds from rocks, supporting the presence of sulfur-oxidizing microorganisms [64]. Furthermore, *Rhodoferax* harbored pathways for dissimilatory nitrate reduction and denitrification (Fig. 3), enabling it to switch electron acceptors and adapt to low-oxygen conditions [8]. This metabolic flexibility could sustain carbon fixation when light is unavailable or in anaerobic conditions.

Interestingly, these carbon-fixing MAGs demonstrated genetic potential to hydrolyze various carbohydrates, such as cellulose, peptidoglycan, and starch, indicating a mixotrophic capacity (Fig. 3 and Supplementary Table S9). The dual ability to combine CO_2_ fixation with organic carbon uptake has also been observed in other environments, such as ocean and geothermal springs [65, 66]. Organic substrates in cryoconite may originate from vegetation at the glacier forefields (Fig. 1). Mixotrophs can shift between autotrophic and heterotrophic metabolism depending on the availability of organic carbon, an adaptive strategy that enhances survival in extreme cryoconite [67].

### Tibetan cryoconite harbor multiple carbon fixation pathways

Carbon fixation occurs through multiple pathways, depending on environmental conditions (Fig. 3 and Fig. 4). The aerobic photosynthetic CBB cycle is the predominant pathway, aligning with previous findings in cryoconites globally [8, 24]. Notably, the aerobic 3-HP bicycle was the second most abundant pathway at metagenome level, despite its absence in MAGs (Fig. 3 and Fig. 4). This pathway, previously unreported in cryoconites, is the dominant carbon fixation pathway in Tibetan glacier foreland [68]. Unlike the CBB cycle which uses CO_2_, the 3-HP bicycle utilizes HCO₃^−^, abundant in cryoconite due to CO₂ dissolution and surrounding carbonates weathering [7]. The prevalence of the 3-HP bicycle might be attributed by the high abundance of Chloroflexota in Tibetan cryoconite, known for harboring genetic potential for this pathway [9, 44].

Aerobic 3-HP/4-HB cycles were detected in the archaeal phylum Thermoproteota, including MAG7 (*Metallosphaera*) and MAG149 (*Saccharolobus*) (Fig. 3). Their genetic potential of 3-HP/4-HB cycle has been previously reported in hot springs [69]. This pathway, also reliant on HCO_3_^−^, is recognized as an energy-efficient carbon fixation mechanism, making it advantageous in nutrient-limited environments [70].

Additionally, oxygen-sensitive pathways, including the DC/4-HB cycles, rTCA cycle, and WL pathway, were also identified, though with low relative abundances (Fig. 4). The presence of these pathways is consistent with depth-dependent oxygen variations within cryoconite, where aerobic conditions dominate the surface, transitioning to microaerobic or anaerobic conditions at greater depths [71, 72]. The structural characteristics of cryoconite particles further contribute to the creation of microaerobic or anaerobic environments within particle interiors [15, 72]. Seasonal variations also influence oxygen concentrations in cryoconite. For example, during winter, ice and snow cover isolate cryoconite from atmospheric oxygen, favoring anaerobic carbon fixation pathways [73, 74].

**Figure 5:**
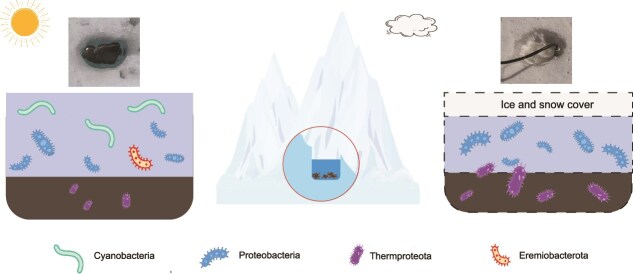
Changes of carbon-fixing microbial communities in response to environmental conditions. Conceptual model illustrates the shifts of carbon-fixing microbial communities in the cryoconite under varying environmental conditions. During light and ice-free periods, all kinds of carbon-fixing microorganisms can coexist, including members belonging to Cyanobacteria, Proteobacteria, Eremiobacterota, and Thermoproteota (the left image). Conversely, under dark and anaerobic conditions, metabolically flexible anaerobic and chemoautotrophic microorganisms become dominant, including members belonging to Thermoproteota and Proteobacteria (the right image).

Based on the metabolic characteristics of carbon-fixing MAGs, a conceptual model was proposed to illustrate microbial community shifts under varying cryoconite conditions (Fig. 5). During light and ice-free periods, various carbon-fixing microorganisms can coexist, including members belonging to Cyanobacteria, Proteobacteria, Eremiobacterota, and Thermoproteota. Conversely, under dark and anaerobic conditions, metabolically flexible anaerobic and chemoautotrophic microorganisms become dominant, including members belonging to Thermoproteota and Proteobacteria.

The presence of diverse carbon fixation microorganisms with multiple pathways reflects the remarkable metabolic versatility of microbial communities in cryoconite. This adaptability enables them to sustain carbon fixation under fluctuating light, oxygen, and temperature conditions, ensuring the persistence of life and ecosystem functioning in one of Earth’s most challenging habitats.

## Conclusion

This study highlights the remarkable diversity of carbon-fixing microorganisms in Tibetan cryoconite and their multiple strategies for carbon fixation. We identified 12 carbon-fixing MAGs across various taxa, including both well-known and previously unclassified genera. Our findings reveal that Cyanobacteria and Proteobacteria play pivotal roles in carbon fixation in cryoconite, supported by their metabolic activity confirmed through DNA-SIP experiments. Additionally, our research uncovers the presence of autotrophic microorganisms with diverse energy utilization capabilities, ranging from phototrophy to chemoautotrophy and atmospheric chemosynthesis.

The discovery of multiple carbon fixation pathways, including the CBB cycle, 3-HP bicycle, and others, underscores the metabolic flexibility of these microbial communities in response to the dynamic environmental conditions of cryoconite. The interaction between various carbon fixation mechanisms and environmental factors such as oxygen levels, light availability, and external microbial inputs further shapes the ecological roles of these microorganisms.

Overall, our study expands the current understanding of carbon cycling in glacial ecosystems, emphasizing the complexity and adaptability of microbial life in cryoconite. These findings provide a foundation for future research on the ecological significance of alternative carbon fixation pathways and their contributions to the broader carbon cycle in cold environments.

## Supplementary Material

SM_Figures_new_ycaf056

SM_Tables_new_ycaf056

Supplementary_Method_new_ycaf056

## Data Availability

The datasets analyzed during the current study are available in the NCBI repository in the accession number of projects PRJNA813429 (https://www.ncbi.nlm.nih.gov/bioproject/PRJNA813429) and PRJNA1101604 (https://www.ncbi.nlm.nih.gov/bioproject/PRJNA1101604).

## References

[1] Wadham JL, Hawkings JR, Tarasov L et al. Ice sheets matter for the global carbon cycle. *Nat Commun* 2019;10:3567. 31417076 10.1038/s41467-019-11394-4 PMC6695407

[2] Sejr MK, Bruhn A, Dalsgaard T et al. Glacial meltwater determines the balance between autotrophic and heterotrophic processes in a Greenland fjord. *Proc Natl Acad Sci USA* 2022;119:e2207024119. 10.1073/pnas.2207024119 36534802 PMC9907075

[3] Wynn PM, Hodson AJ, Heaton THE et al. Nitrate production beneath a high Arctic glacier. *Svalbard Chemical Geology* 2007;244:88–102. 10.1016/j.chemgeo.2007.06.008

[4] Anesio AM, Sattler B, Foreman C et al. Carbon fluxes through bacterial communities on glacier surfaces. *Ann Glaciol* 2010;51:32–40.

[5] Franzetti A, Navarra F, Tagliaferri I et al. Temporal variability of bacterial communities in cryoconite on an alpine glacier. *Environ Microbiol Rep* 2017;9:71–8. 10.1111/1758-2229.12499 27897429

[6] Musilova M, Tranter M, Bennett SA et al. Stable microbial community composition on the Greenland ice sheet. *Front Microbiol* 2015;6:193. 10.3389/fmicb.2015.00193 25852658 PMC4367435

[7] Hugler M, Sievert SM. Beyond the Calvin cycle: autotrophic carbon fixation in the ocean. *Annu Rev Mar Sci* 2011;3:261–89. 10.1146/annurev-marine-120709-142712 21329206

[8] Murakami T, Takeuchi N, Mori H et al. Metagenomics reveals global-scale contrasts in nitrogen cycling and cyanobacterial light-harvesting mechanisms in glacier cryoconite. *Microbiome* 2022;10:50. 10.1186/s40168-022-01238-7 35317857 PMC8941735

[9] Liu Y, Vick-Majors TJ, Priscu JC et al. Biogeography of cryoconite bacterial communities on glaciers of the Tibetan plateau. *FEMS Microbiol Ecol* 2017;93:fix072. 10.1093/femsec/fix072 28531262

[10] Pittino F, Zawierucha K, Poniecka E et al. Functional and taxonomic diversity of anaerobes in supraglacial microbial communities. *Microbiol Spectr* 2023a;11:e0100422. 36939373 10.1128/spectrum.01004-22 PMC10100660

[11] Stibal M, Schostag M, Cameron KA et al. Different bulk and active bacterial communities in cryoconite from the margin and interior of the Greenland ice sheet. *Environ Microbiol Rep* 2015;7:293–300. 10.1111/1758-2229.12246 25405749

[12] Segawa T, Yonezawa T, Edwards A et al. Biogeography of cryoconite forming cyanobacteria on polar and Asian glaciers. *J Biogeogr* 2017;44:2849–61. 10.1111/jbi.13089

[13] Esser M, Ankley P, Aubry-Wake C et al. A preliminary investigation of microbial communities on the Athabasca glacier within deposited organic matter. *Env Sci Adv* 2024;3:355–65. 10.1039/D3VA00176H

[14] Pittino F, Ambrosini R, Seeger M et al. Geographical variability of bacterial communities of cryoconite holes of Andean glaciers. *Sci Rep* 2023b;13:2633. 10.1038/s41598-022-24373-5 36788266 PMC9929092

[15] Segawa T, Takeuchi N, Mori H et al. Redox stratification within cryoconite granules influences the nitrogen cycle on glaciers. *FEMS Microbiol Ecol* 2020;96:fiaa199. 10.1093/femsec/fiaa199 32990745

[16] Trivedi CB, Stamps BW, Lau GE et al. Microbial metabolic redundancy is a key mechanism in a sulfur-rich glacial ecosystem. *Msystems* 2020;5:00504–20. 10.1128/mSystems.00504-20 PMC7406229 32753510

[17] Dumont MG, Murrell JC. Stable isotope probing—linking microbial identity to function. *Nat Rev Microbiol* 2005;3:499–504. 10.1038/nrmicro1162 15886694

[18] Neufeld JD, Vohra J, Dumont MG et al. DNA stable-isotope probing. *Nat Protoc* 2007;2:860–6. 17446886 10.1038/nprot.2007.109

[19] Coskun ÖK, Gomez-Saez GV, Beren M et al. Quantifying genome-specific carbon fixation in a 750-meter deep subsurface hydrothermal microbial community. *FEMS Microbiol Ecol* 2024;100:fiae062. 10.1093/femsec/fiae062 38632042 PMC11075769

[20] Liu Z, Sun Y, Zhang Y et al. Soil microbes transform inorganic carbon into organic carbon by dark fixation pathways in desert soil. *J Geophys Res Biogeosciences* 2021;126:e2020JG006047. 10.1029/2020JG006047

[21] Cotton CAR, Edlich-Muth C, Bar-Even A. Reinforcing carbon fixation: CO_2_ reduction replacing and supporting carboxylation. *Curr Opin Biotechnol* 2018;49:49–56. 10.1016/j.copbio.2017.07.014 28803187

[22] Figueroa IA, Barnum TP, Somasekhar PY et al. Metagenomics-guided analysis of microbial chemolithoautotrophic phosphite oxidation yields evidence of a seventh natural CO_2_ fixation pathway. *Proc Natl Acad Sci USA* 2018;115:E92–101. 10.1073/pnas.1715549114 29183985 PMC5776814

[23] Cameron KA, Hodson AJ, Osborn AM. Carbon and nitrogen biogeochemical cycling potentials of supraglacial cryoconite communities. *Polar Biol* 2012;35:1375–93. 10.1007/s00300-012-1178-3

[24] Franzetti A, Tagliaferri I, Gandolfi I et al. Light-dependent microbial metabolisms drive carbon fluxes on glacier surfaces. *ISME J* 2016;10:2984–8. 10.1038/ismej.2016.72 27128995 PMC5148193

[25] Yao T, Thompson L, Yang W et al. Different glacier status with atmospheric circulations in Tibetan plateau and surroundings. *Nat Clim Chang* 2012;2:663–7. 10.1038/nclimate1580

[26] Liu Y, Ji M, Yu T et al. A genome and gene catalog of glacier microbiomes. *Nat Biotechnol* 2022;40:1341–8. 10.1038/s41587-022-01367-2 35760913

[27] Bolger AM, Lohse M, Usadel B. Trimmomatic: a flexible trimmer for Illumina sequence data. *Bioinformatics* 2014;30:2114–20. 10.1093/bioinformatics/btu170 24695404 PMC4103590

[28] Li D, Liu CM, Luo R et al. MEGAHIT: an ultra-fast single-node solution for large and complex metagenomics assembly via succinct de Bruijn graph. *Bioinformatics* 2015;31:1674–6. 10.1093/bioinformatics/btv033 25609793

[29] Hyatt D, Chen GL, LoCascio PF et al. Prodigal: prokaryotic gene recognition and translation initiation site identification. *BMC Bioinformatics* 2010;11:119. 10.1186/1471-2105-11-119 20211023 PMC2848648

[30] Mirdita M, Steinegger M, Breitwieser F et al. Fast and sensitive taxonomic assignment to metagenomic contigs. *Bioinformatics* 2021;37:3029–31. 10.1093/bioinformatics/btab184 33734313 PMC8479651

[31] Steinegger M, Söding J. MMseqs2 enables sensitive protein sequence searching for the analysis of massive data sets. *Nat Biotechnol* 2017;35:1026–8. 10.1038/nbt.3988 29035372

[32] Patro R, Duggal G, Love MI et al. Salmon provides fast and bias-aware quantification of transcript expression. *Nat Methods* 2017;14:417–9. 10.1038/nmeth.4197 28263959 PMC5600148

[33] Uritskiy GV, DiRuggiero J, Taylor J. MetaWRAP—a flexible pipeline for genome-resolved metagenomic data analysis. *Microbiome* 2018;6:158. 30219103 10.1186/s40168-018-0541-1 PMC6138922

[34] Parks DH, Imelfort M, Skennerton CT et al. CheckM: assessing the quality of microbial genomes recovered from isolates, single cells, and metagenomes. *Genome Res* 2015;25:1043–55. 10.1101/gr.186072.114 25977477 PMC4484387

[35] Olm MR, Brown CT, Brooks B et al. dRep: a tool for fast and accurate genomic comparisons that enables improved genome recovery from metagenomes through de-replication. *The ISME Journal.* 2017;11:2864–8. 10.1038/ismej.2017.126 28742071 PMC5702732

[36] Seemann T . Prokka: rapid prokaryotic genome annotation. *Bioinformatics* 2014;30:2068–9. 10.1093/bioinformatics/btu153 24642063

[37] Li H . Aligning sequence reads, clone sequences and assembly contigs with BWA-MEM. arXiv preprint. 2013;1303:3997.

[38] Danecek P, Bonfield JK, Liddle J et al. Twelve years of SAMtools and BCFtools. *GigaScience* 2021;10:giab008. 10.1093/gigascience/giab008 33590861 PMC7931819

[39] Kanehisa M, Sato Y, Kawashima M et al. KEGG as a reference resource for gene and protein annotation. *Nucleic Acids Res* 2016;44:D457–62. 10.1093/nar/gkv1070 26476454 PMC4702792

[40] Zheng J, Ge Q, Yan Y et al. dbCAN3: automated carbohydrate-active enzyme and substrate annotation. *Nucleic Acids Res* 2023;51:W115–21. 10.1093/nar/gkad328 37125649 PMC10320055

[41] Chaumeil P-A, Mussig AJ, Hugenholtz P et al. GTDB-Tk: a toolkit to classify genomes with the genome taxonomy database. *Bioinformatics* 2020;36:1925–7. 10.1093/bioinformatics/btz848 PMC7703759 31730192

[42] Price MN, Dehal PS, Arkin AP. FastTree: computing large minimum evolution trees with profiles instead of a distance matrix. *Mol Biol Evol* 2009;26:1641–50. 10.1093/molbev/msp077 19377059 PMC2693737

[43] Letunic I, Bork P. Interactive tree of life (iTOL) v5: an online tool for phylogenetic tree display and annotation. *Nucleic Acids Res* 2021;49:W293–6. 10.1093/nar/gkab301 33885785 PMC8265157

[44] Berg IA . Ecological aspects of the distribution of different autotrophic CO_2_ fixation pathways. *Appl Environ Microbiol* 2011;77:1925–36. 10.1128/AEM.02473-10 21216907 PMC3067309

[45] Edgar RC . MUSCLE: a multiple sequence alignment method with reduced time and space complexity. *BMC Bioinformatics* 2004;5:113. 10.1186/1471-2105-5-113 15318951 PMC517706

[46] Bay SK, Dong X, Bradley JA et al. Trace gas oxidizers are widespread and active members of soil microbial communities. *Nat Microbiol* 2021;6:246–56. 10.1038/s41564-020-00811-w 33398096

[47] Chrismas NA, Barker G, Anesio AM et al. Genomic mechanisms for cold tolerance and production of exopolysaccharides in the Arctic cyanobacterium Phormidesmis priestleyi BC1401. *BMC Genomics* 2016;17:533. 10.1186/s12864-016-2846-4 27485510 PMC4971617

[48] Gokul JK, Hodson AJ, Saetnan ER et al. Taxon interactions control the distributions of cryoconite bacteria colonizing a high Arctic ice cap. *Mol Ecol* 2016;25:3752–67. 10.1111/mec.13715 27261672

[49] Antony R, Mongad D, Sanyal A et al. Holed up, but thriving: impact of multitrophic cryoconite communities on glacier elemental cycles. *Sci Total Environ* 2024;933:173187. 10.1016/j.scitotenv.2024.173187 38750762

[50] Gladkov G, Kimeklis A, Tembotov R et al. The effect of geographic location and physiochemical characteristics on the cryoconite prokaryotic communities from the Arctic, Antarctic, and central Caucasus regions. *Sci Rep* 2024;14:15838. 38982048 10.1038/s41598-024-64452-3 PMC11233692

[51] Zhang Z, Liu Y, Zhao W et al. Radiation impacts gene redundancy and biofilm regulation of cryoconite microbiomes in northern hemisphere glaciers. *Microbiome* 2023;11:228. 37848997 10.1186/s40168-023-01621-y PMC10583317

[52] Qi J, Ji M, Wang W et al. Effect of Indian monsoon on the glacial airborne bacteria over the Tibetan plateau. *Sci Total Environ* 2022;831:154980. 10.1016/j.scitotenv.2022.154980 35378188

[53] Dunham EC, Dore JE, Skidmore ML et al. Lithogenic hydrogen supports microbial primary production in subglacial and proglacial environments. *Proc Natl Acad Sci USA* 2021;118:e2007051117. 10.1073/pnas.2007051117 33419920 PMC7812807

[54] Ji M, Williams TJ, Montgomery K et al. Candidatus Eremiobacterota, a metabolically and phylogenetically diverse terrestrial phylum with acid-tolerant adaptations. *ISME J* 2021;15:2692–707. 10.1038/s41396-021-00944-8 33753881 PMC8397712

[55] Lin Y, Wang L, Xu K et al. Revealing taxon-specific heavy metal-resistance mechanisms in denitrifying phosphorus removal sludge using genome-centric metaproteomics. *Microbiome* 2021;9:67. 10.1186/s40168-021-01016-x 33752740 PMC7986553

[56] Bowers RM, Kyrpides NC, Stepanauskas R et al. Minimum information about a single amplified genome (MISAG) and a metagenome-assembled genome (MIMAG) of bacteria and archaea. *Nat Biotechnol* 2017;35:725–31. 10.1038/nbt.3893 28787424 PMC6436528

[57] Garritano AN, Song W, Thomas T. Carbon fixation pathways across the bacterial and archaeal tree of life. *PNAS Nexus* 2022;1:pgac226. 10.1093/pnasnexus/pgac226 36712370 PMC9802188

[58] Baker JM, Riester CJ, Skinner BM et al. Genome sequence of Rhodoferax antarcticus ANT.BR^T^; a psychrophilic purple nonsulfur bacterium from an Antarctic microbial mat. *Microorganisms* 2017;5:8. 10.3390/microorganisms5010008 28230808 PMC5374385

[59] Xu Y, Teng Y, Dai S et al. Atmospheric trace gas oxidizers contribute to soil carbon fixation driven by key soil conditions in terrestrial ecosystems. *Environ Sci Technol* 2024;58:21617–28. 10.1021/acs.est.4c06516 39443297

[60] Cordero PRF, Bayly K, Man Leung P et al. Atmospheric carbon monoxide oxidation is a widespread mechanism supporting microbial survival. *ISME J* 2019;13:2868–81. 10.1038/s41396-019-0479-8 31358912 PMC6794299

[61] Fang Y, Liu J, Yang J et al. Compositional and metabolic responses of autotrophic microbial community to salinity in lacustrine environments. *mSystems* 2022;7:e0033522. 10.1128/msystems.00335-22 35862818 PMC9426519

[62] Haan D, Zuo Y, Gros V et al. Photochemical production of carbon monoxide in snow. *J Atmos Chem* 2001;40:217–30. 10.1023/A:1012216112683

[63] Xie H, Zafiriou OC. Evidence for significant photochemical production of carbon monoxide by particles in coastal and oligotrophic marine waters. *Geophys Res Lett* 2009;36:L23606.

[64] Torres MA, Moosdorf N, Hartmann J et al. Glacial weathering, sulfide oxidation, and global carbon cycle feedbacks. *Proc Natl Acad Sci USA* 2017;114:8716–21. 10.1073/pnas.1702953114 28760954 PMC5565423

[65] Qi Y, Chen Y, Xie Y et al. Analysis of nearly 3000 archaeal genomes from terrestrial geothermal springs sheds light on interconnected biogeochemical processes. *Nat Commun* 2024;15:4066. 10.1038/s41467-024-48498-5 38744885 PMC11094006

[66] Zhao Y, Liu P, Rui J et al. Dark carbon fixation and chemolithotrophic microbial community in surface sediments of the cascade reservoirs, Southwest China. *Sci Total Environ* 2020;698:134316. 10.1016/j.scitotenv.2019.134316 31783464

[67] Taubert M, Overholt WA, Heinze BM et al. Bolstering fitness via CO_2_ fixation and organic carbon uptake: mixotrophs in modern groundwater. *ISME J* 2022;16:1153–62. 10.1038/s41396-021-01163-x 34876683 PMC8941145

[68] Zhang J, Ma A, Zhou H et al. Unexpected high carbon losses in a continental glacier foreland on the Tibetan plateau. *ISME Commun* 2022;2:68. 10.1038/s43705-022-00148-x 37938688 PMC9723710

[69] Lin KH, Liao BY, Chang HW et al. Metabolic characteristics of dominant microbes and key rare species from an acidic hot spring in Taiwan revealed by metagenomics. *BMC Genomics* 2015;16:1029. 10.1186/s12864-015-2230-9 26630941 PMC4668684

[70] Berg IA, Kockelkorn D, Buckel W et al. A 3-Hydroxypropionate/4-Hydroxybutyrate autotrophic carbon dioxide assimilation pathway in archaea. *Science* 2007;318:1782–6. 10.1126/science.1149976 18079405

[71] Buda J, Poniecka EA, Rozwalak P et al. Is oxygenation related to the decomposition of organic matter in cryoconite holes? *Ecosystems* 2022;25:1510–21. 10.1007/s10021-021-00729-2

[72] Poniecka EA, Bagshaw EA, Tranter M et al. Rapid development of anoxic niches in supraglacial ecosystems. *Arct Antarct Alp Res* 2018;50:S100015. 10.1080/15230430.2017.1420859

[73] Fountain AG, Tranter M, Nylen TH et al. Evolution of cryoconite holes and their contribution to meltwater runoff from glaciers in the McMurdo dry valleys. *Antarct J Glaciol* 2004;50:35–45. 10.3189/172756504781830312

[74] Poniecka EA, Bagshaw EA, Sass H et al. Physiological capabilities of cryoconite hole microorganisms. *Front Microbiol* 2020;11:1783. 32849402 10.3389/fmicb.2020.01783 PMC7412143

