# Supplementary material for: Metabolic diversity and adaptation of carbon-fixing microorganisms in extreme glacial cryoconite
Source: ISME Commun. 2024 Mar 30;5(1):ycaf056. doi: 10.1093/ismeco/ycaf056 (PMC12011081; doi:10.1093/ismeco/ycaf056)
Supplement: SM_Figures_new_ycaf056 [file sm_figures_new_ycaf056.docx]

**Supplementary Information**

**Metabolic Diversity and Adaptation of Carbon-Fixing Microorganisms in** **Extreme Glacial Cryoconite**

Yuying Chen^1^, Yongqin Liu^1,2,5,*^, Mukan Ji^1^, Zhihao Zhang^2^, Tingting Xing^2^, Hongan Pan^3^, Keshao Liu^2^, Yueang Li^4^, Penfei Liu^1^

^1^Center for the Pan-Third Pole Environment, Lanzhou University, Lanzhou, 730000, China

^2^State Key Laboratory of Tibetan Plateau Earth System, Environment and Resources (TPESER), Institute of Tibetan Plateau Research, Chinese Academy of Sciences, Beijing 100101, China

^3^Laboratory of Soil Microbial Geography, School of Geographical Sciences, Nanjing Normal University, Nanjing, 210023, China

^4^Faculty of Forestry, Natural Resource Conservation, University of British Columbia, 2300 West Mall, Vancouver BC V6T 1Z4, Canada

^5^University of Chinese Academy of Sciences, Beijing, 100049, China

*Corresponding author:

Yongqin Liu, [yql@lzu.edu.cn](mailto:yql@lzu.edu.cn)

Center for the Pan-third Pole Environment, Lanzhou University

222 South Tianshui Road, Lanzhou, Gansu Province, 730000, P.R.China

**Running title:** Carbon-Fixing Microorganisms


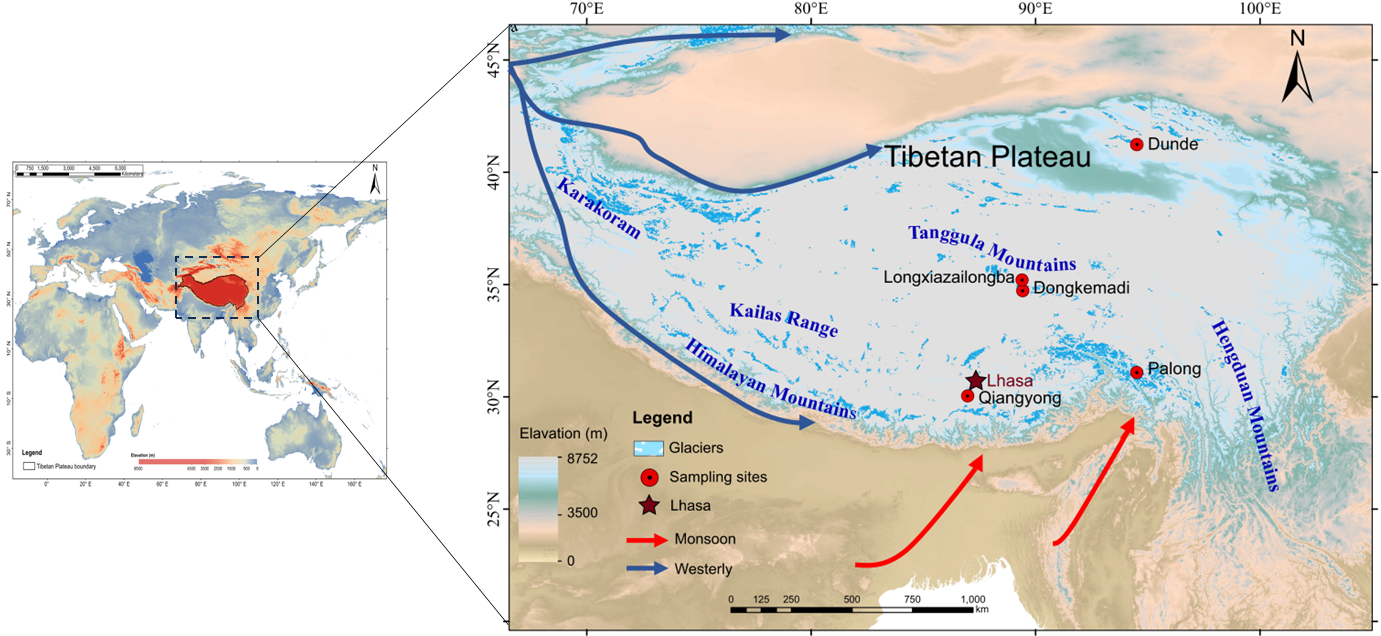


**Figure S1: Location map of the five sampled glaciers on the Tibetan Plateau.** The left panel shows the location of the Tibetan Plateau on a global map. The right panel details the sampling sites across the Tibetan Plateau. Blue and red arrows indicate the Westerly and the Indian Monsoon, respectively.


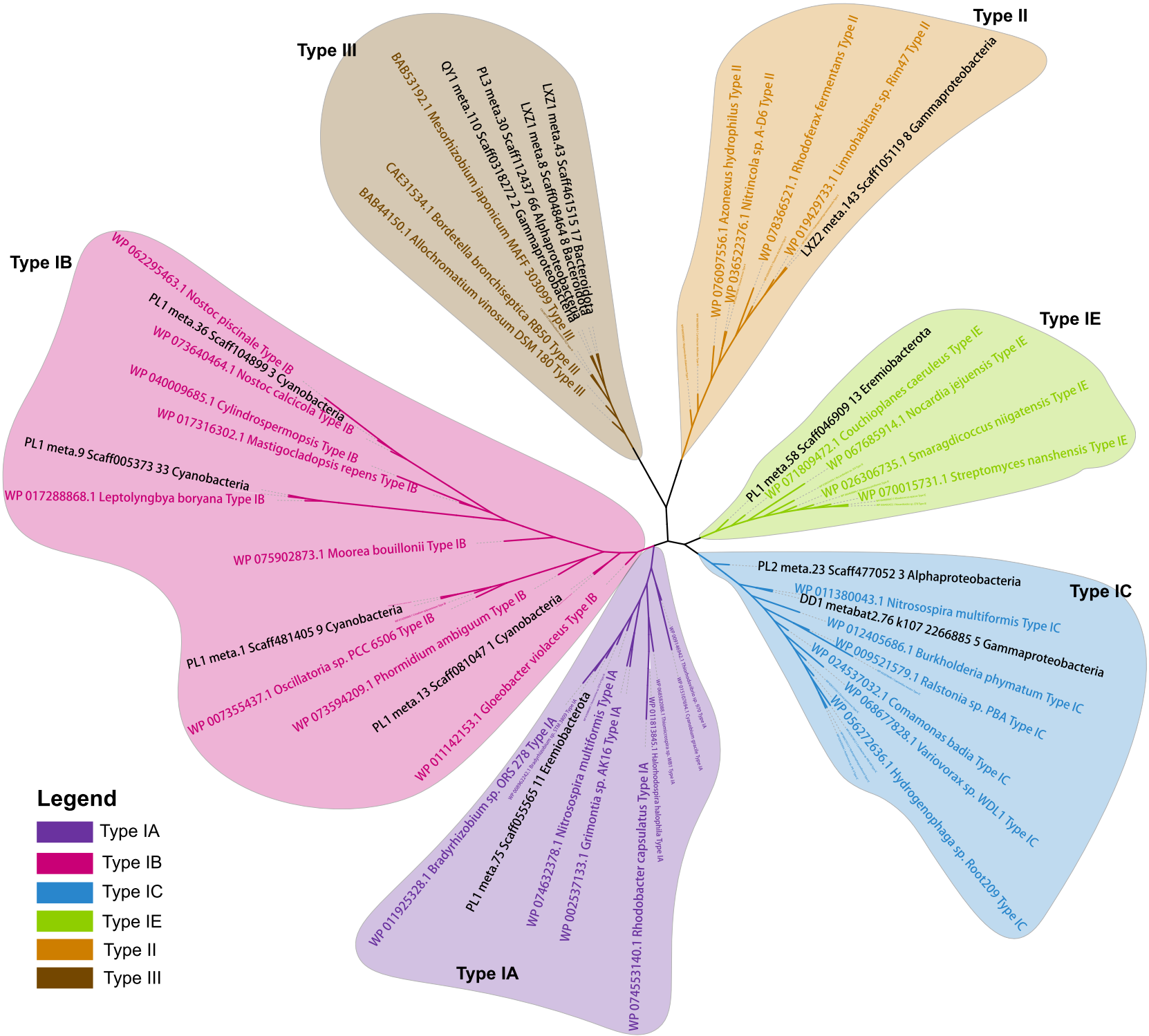


**Figure S2: Phylogenetic tree of** **RuBisCO large subunit amino acid sequences (rbcL).** Each color represents a different type of Rubisco enzyme. Sequences identified in this study are shown in black.


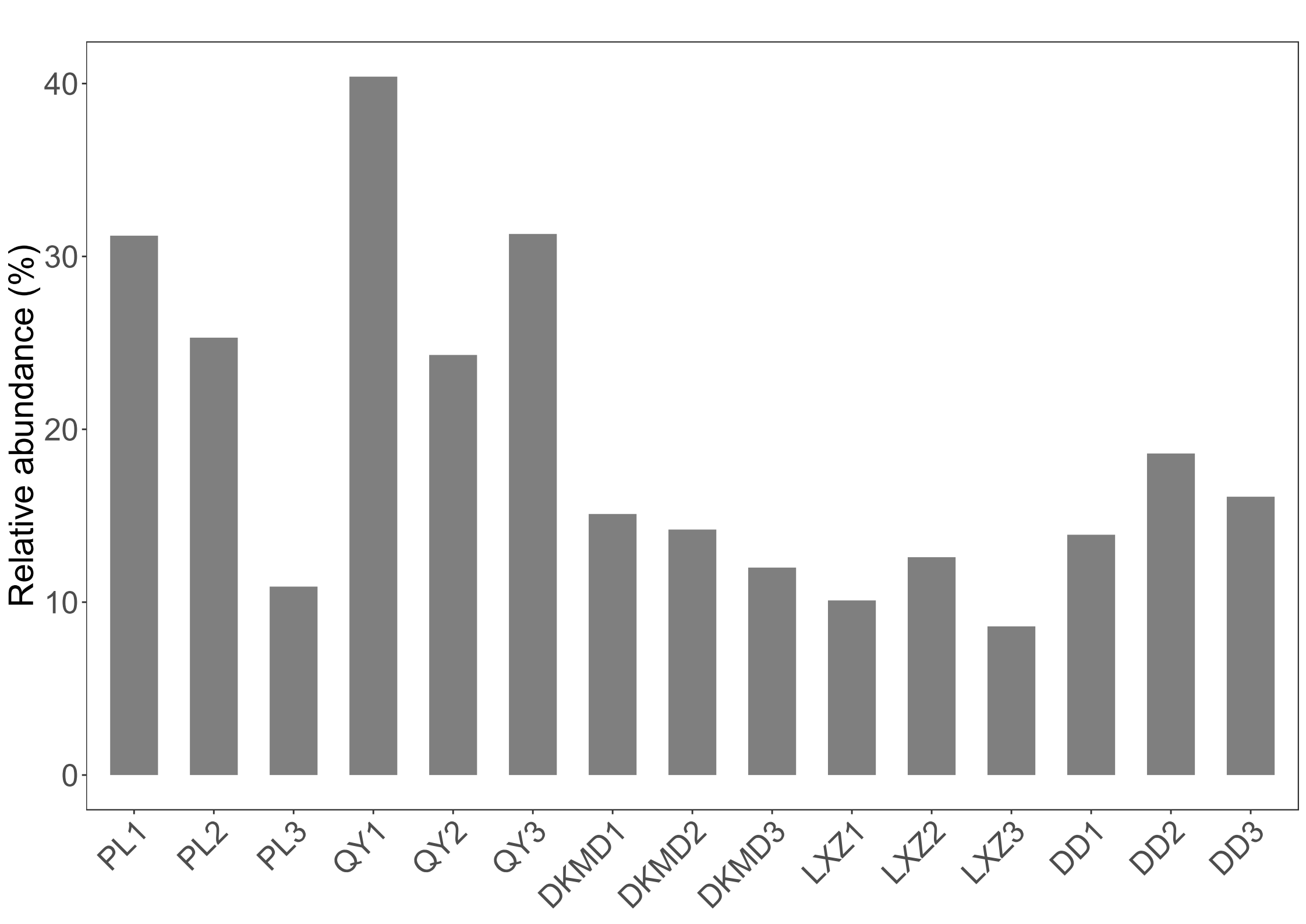
**Figure S3: Relative abundance of potential carbon fixation MAGs across five glaciers on the Tibetan Plateau.**


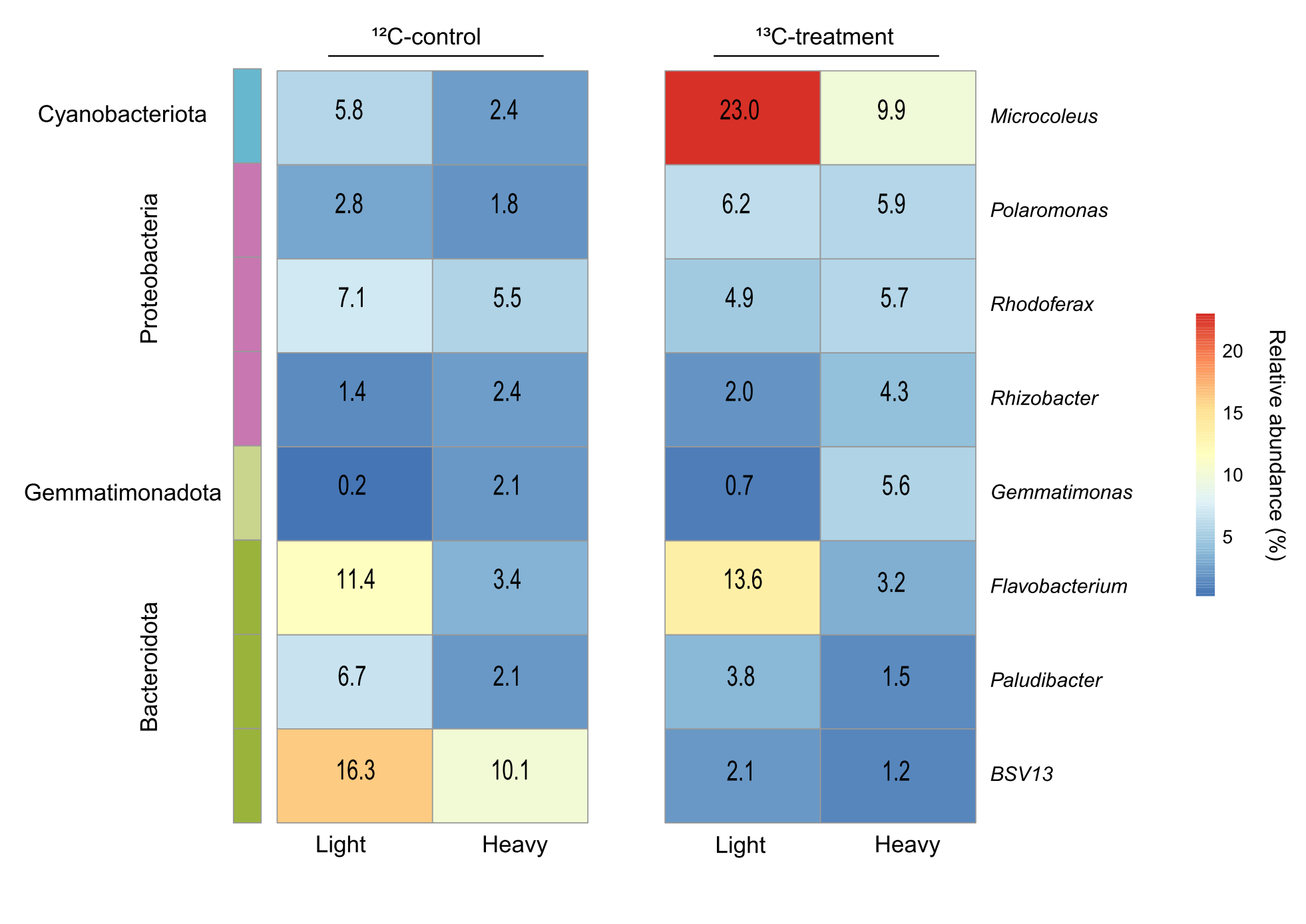
 **Figure S4: Relative abundance of each genus in the heavy and light fractions on Day 20.**


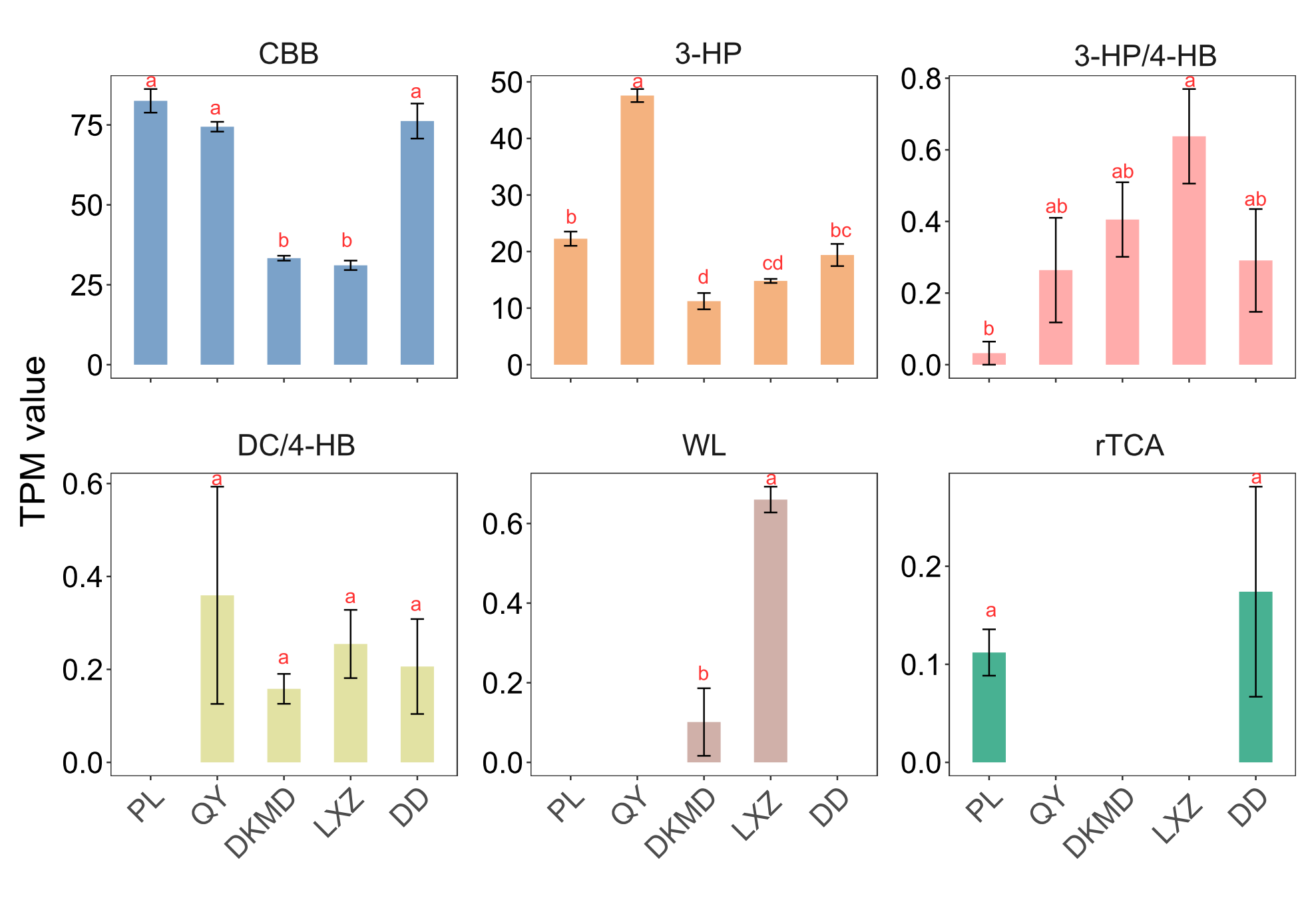
**Figure S5: Comparison of TPM value of carbon fixation pathways across five glaciers at metagenomic level.** Different carbon fixation pathways are distinguished by colors of the graph. The horizontal axis represents different glaciers, while the vertical axis represents the TPM value. The height of each bar indicates the average TPM value of the carbon fixation pathway in the corresponding glacier. Error bars depict the range of measurements from three replicate samples. Different letters represent significant differences at *P* < 0.05 (one-way ANOVA with Tukey's post hoc test).
