# Supplementary material for: Metabolic diversity and adaptation of carbon-fixing microorganisms in extreme glacial cryoconite
Source: ISME Commun. 2024 Mar 30;5(1):ycaf056. doi: 10.1093/ismeco/ycaf056 (PMC12011081; doi:10.1093/ismeco/ycaf056)
Supplement: Supplementary_Method_new_ycaf056 [file supplementary_method_new_ycaf056.docx]

**Supplementary Method**

**Metabolic Diversity and Adaptation of Carbon-Fixing Microorganisms in** **Extreme Glacial Cryoconite**

Yuying Chen^1^, Yongqin Liu^1,2,5,*^, Mukan Ji^1^, Zhihao Zhang^2^, Tingting Xing^2^, Hongan Pan^3^, Keshao Liu^2^, Yueang Li^4^, Penfei Liu^1^

^1^Center for the Pan-Third Pole Environment, Lanzhou University, Lanzhou, 730000, China

^2^State Key Laboratory of Tibetan Plateau Earth System, Environment and Resources (TPESER), Institute of Tibetan Plateau Research, Chinese Academy of Sciences, Beijing 100101, China

^3^Laboratory of Soil Microbial Geography, School of Geographical Sciences, Nanjing Normal University, Nanjing, 210023, China

^4^Faculty of Forestry, Natural Resource Conservation, University of British Columbia, 2300 West Mall, Vancouver BC V6T 1Z4, Canada

^5^University of Chinese Academy of Sciences, Beijing, 100049, China

*Corresponding author:

Yongqin Liu, [yql@lzu.edu.cn](mailto:yql@lzu.edu.cn)

Center for the Pan-third Pole Environment, Lanzhou University

222 South Tianshui Road, Lanzhou, Gansu Province, 730000, P.R.China

**Running title:** Carbon-Fixing Microorganisms

**Sample collection**

All sampled cryoconite holes were located at the glacier termini and were free of ice cover during the summer collection. The specific procedure for sample collection involved the randomly selection of three cryoconite holes as biological replicates. To ensure sterility, sterilized syringes were used to collect the overlying water from the cryoconite holes. Additionally, an autoclaved-sterilized spoon was employed to collect sediment samples from the bottom of the cryoconite holes. The sediment samples were then carefully placed into pre-cleaned 500 mL Nalgene bottles (Nalgene Nunc International, St. Louis, Mo., USA) along with the water samples from the cryoconite holes. All Nalgene bottles used for sampling were subjected to pre-treatment in the laboratory. This involved rinsing them with Milli-Q water, autoclaving at 120°C for 15 minutes, and then drying at 55°C. After collection, all samples were promptly placed in a thermos box and stored in a dark environment. Within a maximum time frame of 4 hours, the samples were transported to the researchers' tents and maintained frozen at -20°C until they were ready for laboratory analysis.

**DNA extrection**

Cryoconite sample (0.5 g) was immersed in 1.5 mL of lysis buffer (a mixture of 20 mg mL^-1^ proteinase K, 0.1 M EDTA, and 10% SDS) and then incubated at 55°C for 2 hours. DNA extraction was then performed using the FastDNA®Spin Kit for Soil (MP Biomedicals, Santa Ana, CA, USA) according to the manufacturer's instructions. The extracted DNA was eluted into 100 µL of TE buffer. DNA gel electrophoresis analysis was conducted using a 1% (w/v) agarose gel. The DNA bands were excised from the gel and purified using the Gel Purification Kit (TaKaRa, Japan). The concentration and purity of the extracted DNA were measured using a NanoDrop 1000 spectrophotometer (Thermo-Scientific, Wilmington, DE, USA). The extracted DNA was stored at -80°C until amplification and sequencing.

**Metagenomic library construction and sequencing**

DNA from all 15 cryoconite samples, each with a quantity of 100 ng, was fragmented using an ultrasonic disruptor (Bioruptor™ sonicator, Diagenode, Belgium), resulting in fragments ranging from 100 to 400 bp in length. The fragmented DNA was used to construct a metagenomic library with fragment sizes of 300 to 500 bp using the KAPA HyperPrep Kit (Roche, Switzerland). After library construction, the library was quantified using the Qubit dsDNA HS Assay Kit (Invitrogen, USA) on the Qubit 2.0 fluorometer (Invitrogen, USA). Following quantification, the library was diluted to a concentration of 1 ng μL^-1^, and the insert fragments in the library were detected using an Agilent 2100 Bioanalyzer (Agilent Technologies, Germany) with the Agilent High Sensitivity DNA kit (Agilent Technologies, Germany). If the sizes of the insert fragments in the library matched the expected range, the library's effective concentration was then accurately determined using quantitative Polymerase Chain Reaction (PCR), ensuring an effective concentration greater than 2 nM. The metagenomic sequencing for this study was performed by MAGIGENE (Shenzhen, China) using the Illumina HiSeq 2500 platform PE150 with 150-bp paired-end reads.

**Experimental conditions for DNA-SIP**

Due to the limited sample volumes from other glaciers, cryoconite from the Longxiazailongba (LXZ) glacier was chosen for DNA-SIP. Based on monitoring cryoconite temperatures at the LXZ glacier over two consecutive months, we revealed that the microenvironment within cryoconite holes can reach up to 15°C at the peak time of the day (**Figure 1**). Considering that the growth temperature range for cryoconite microorganisms is approximately 1-20°C, with the optimal range for most between 15 and 20°C (Margesin *et al.*, 2003, Zhang *et al.*, 2011, Schumann *et al.*, 2012, Zhang *et al.*, 2012, Yang *et al.*, 2021), and that many enzyme and microbial activity assays are easier to detect and measure at 15°C, all cultures were incubated at 15°C with illumination levels of 2000–2500 lx. Given that sediment depth affects light exposure, to minimize self-shading, only surface sediment samples (<1 cm) were collected for DNA extraction and ^13^C value determination.

**Determination of δ^13^C of total organic carbon**

To determine the δ^13^C of total organic carbon (δ^13^C-TOC), sediment samples from the cultivation (~0.3-0.5 g wet weight/tube) were lyophilized and milled into fine particles. Subsequently, 15-20 mg of each sample was weighed, loaded into silver boats, and steamed with concentrated hydrochloric acid in a drying vessel for 12 hours to remove inorganic carbon. The samples were then dried overnight at 50 °C. Using an elemental analyzer (Vario El Ⅲ/Isoprime, Germany) quantified the δ^13^C, with three independent replicates performed for each sample.

**DNA extraction, gradient centrifugation and fractionation**

Based on the δ^13^C of total organic carbon, values increased to their peak and then stabilized on the 20th day. To minimize cross-feeding caused by prolonged cultivation, total DNA was extracted from samples collected on the 20th day using a FastDNA®Spin Kit for Soil (MP Biomedicals, Santa Ana, CA, USA) according to the manufacturer's instructions and quantified using a NanoDrop spectrophotometer (Thermo-Scientific, USA). For gradient centrifugation, 2 μg of sample DNA was thoroughly mixed with cesium chloride (CsCl) solution into a 5 mL system, and the buoyant density was adjusted to 1.725 g mL^−1^. The mixture was transferred to a 5.1 mL ultracentrifugation tube, placed in a VTi 65.2 vertical rotor (Beckman Coulter, Krefeld, Germany), and centrifuged at 20°C for 44 hours at 190,000×g in a Hitachi Preparative Centrifuge CP80WX (Hitachi Ultras, Japan). After centrifugation, the gradient solution was fractionated from bottom to top into 11 DNA fractions, displaced with sterile water using a NE-1000 single syringe pump (New Era Pump Systems, Inc., USA). Each fraction contained approximately 0.5 mL of solution. The buoyant density of each collected fraction was determined using an AR200 digital hand-held refractometer (Reichert, USA). After which, DNA was precipitated from CsCl gradient fractions using polyethylene glycol and dissolved in 30 μL of elution buffer.

**Quantitative PCR, PCR amplification and high-throughput sequencing**

The universal primers 515F (5’-GTGCCAGCMGCCGCGGTAA-3’) and 907R (5’-CCGTCAATTCMTTTRAGTTT-3’) were used for the amplification of the 16S rRNA gene for Quantitative PCR (qPCR) (Angenent *et al.*, 2005). Each reaction (25 μL) consisted of 12.5 μL of 2× SYBR Premix Ex Taq (TaKaRa Biotechnology, Japan), 0.5 μL of each primer (10 μM), 2 μL of template DNA, and 9.5 μL of double-distilled water (ddH2O, sigma, USA). The qPCR was carried out on an ABI QauntStudio Real-Time Detection System (Applied Biosystems, USA) using the following cycling conditions: 94°C for 3 min and then 30 PCR cycles at 94°C for 45 sec, 58°C for 60 sec, and 72°C for 60 sec, followed by a melt curve from 58°C to 94°C at a 0.5°C increment. Standard curves were developed using 10-fold serial dilutions of plasmid DNA ranging from 10^2^ to 10^8^ copies μL^-1^. The qPCR amplification efficiency ranged from 90% to 100%, with an R^2^ of 0.99 to 1.

Barcoded PCR amplification of the 16S rRNA gene from the heavy and light fractions was conducted using bacterial primers 515F (5’-GTGCCAGCMGCCGCGGTAA-3’) and 907R (5’-CCGTCAATTCMTTTRAGTTT-3’) (Angenent *et al.*, 2005). The heavy fractions exhibited buoyant densities ranging from ~1.71 g mL^-1^ to ~1.73 g mL^-1^, while the light fractions ranged from ~1.67 g mL^-1^ to ~1.69 g mL^-1^, with an increased buoyant density of ~0.04 g mL^-1^ (Lueders *et al.*, 2004). The library was sequenced on an Illumina MiSeq platform using standard procedures for a 2 × 300 cycle combination mode at Meiji Biotechnology Company (Shanghai, China).

**MiSeq sequence analysis**

Raw sequence data were processed using MOTHUR v1.34.3 (Schloss Patrick *et al.*, 2009). Paired-end reads were merged and aligned against the Silva reference alignment database (Silva, release 138), which was trimmed to include only the amplified region while removing sequences that did not align. Chimeric sequences were identified using UCHIME (Edgar *et al.*, 2011) and subsequently removed. The remaining sequences were classified using the Bayesian classifier against the Silva database (release 138), with a minimum confidence score of 80% (Wang *et al.*, 2007). All Eukaryota, chloroplasts, mitochondria, and unclassified sequences were removed. Finally, sequences were classified into Amplicon Sequence Variants (ASVs) at 100% identity, and singletons were removed. The dataset was sub-sampled to an equal size of 14,085 sequences, corresponding to the smallest sample size across the entire dataset.

**Reference:**

Angenent LT, Kelley ST, St Amand A, Pace NR & Hernandez MT (2005) Molecular identification of potential pathogens in water and air of a hospital therapy pool. *Proc Natl Acad Sci U S A* **102**: 4860-4865.

Edgar RC, Haas BJ, Clemente JC, Quince C & Knight R (2011) UCHIME improves sensitivity and speed of chimera detection. *Bioinformatics* **27**: 2194-2200.

Lueders T, Manefield M & Friedrich MW (2004) Enhanced sensitivity of DNA- and rRNA-based stable isotope probing by fractionation and quantitative analysis of isopycnic centrifugation gradients. *Environ Microbiol* **6**: 73-78.

Margesin R, Spröer C, Schumann P & Schinner F (2003) Pedobacter cryoconitis sp. nov., a facultative psychrophile from alpine glacier cryoconite. *Int J Syst Evol Microbiol* **53**: 1291-1296.

Schloss Patrick D, Westcott Sarah L, Ryabin T*, et al.* (2009) Introducing mothur: Open-Source, Platform-Independent, Community-Supported Software for Describing and Comparing Microbial Communities. *Applied and Environmental Microbiology* **75**: 7537-7541.

Schumann P, Zhang DC, Redzic M & Margesin R (2012) Alpinimonas psychrophila gen. nov., sp. nov., an actinobacterium of the family Microbacteriaceae isolated from alpine glacier cryoconite. *Int J Syst Evol Microbiol* **62**: 2724-2730.

Wang Q, Garrity George M, Tiedje James M & Cole James R (2007) Naïve Bayesian Classifier for Rapid Assignment of rRNA Sequences into the New Bacterial Taxonomy. *Applied and Environmental Microbiology* **73**: 5261-5267.

Yang LL, Pang Y, Liu HC, Xin YH & Liu Q (2021) Mucilaginibacter glaciei sp. nov. and Mucilaginibacter pankratovii sp. nov., isolated from a glacier on the Tibetan Plateau. *Int J Syst Evol Microbiol* **71**.

Zhang DC, Busse HJ, Liu HC, Zhou YG, Schinner F & Margesin R (2011) Sphingomonas glacialis sp. nov., a psychrophilic bacterium isolated from alpine glacier cryoconite. *Int J Syst Evol Microbiol* **61**: 587-591.

Zhang DC, Schumann P, Redzic M, Zhou YG, Liu HC, Schinner F & Margesin R (2012) Nocardioides alpinus sp. nov., a psychrophilic actinomycete isolated from alpine glacier cryoconite. *Int J Syst Evol Microbiol* **62**: 445-450.


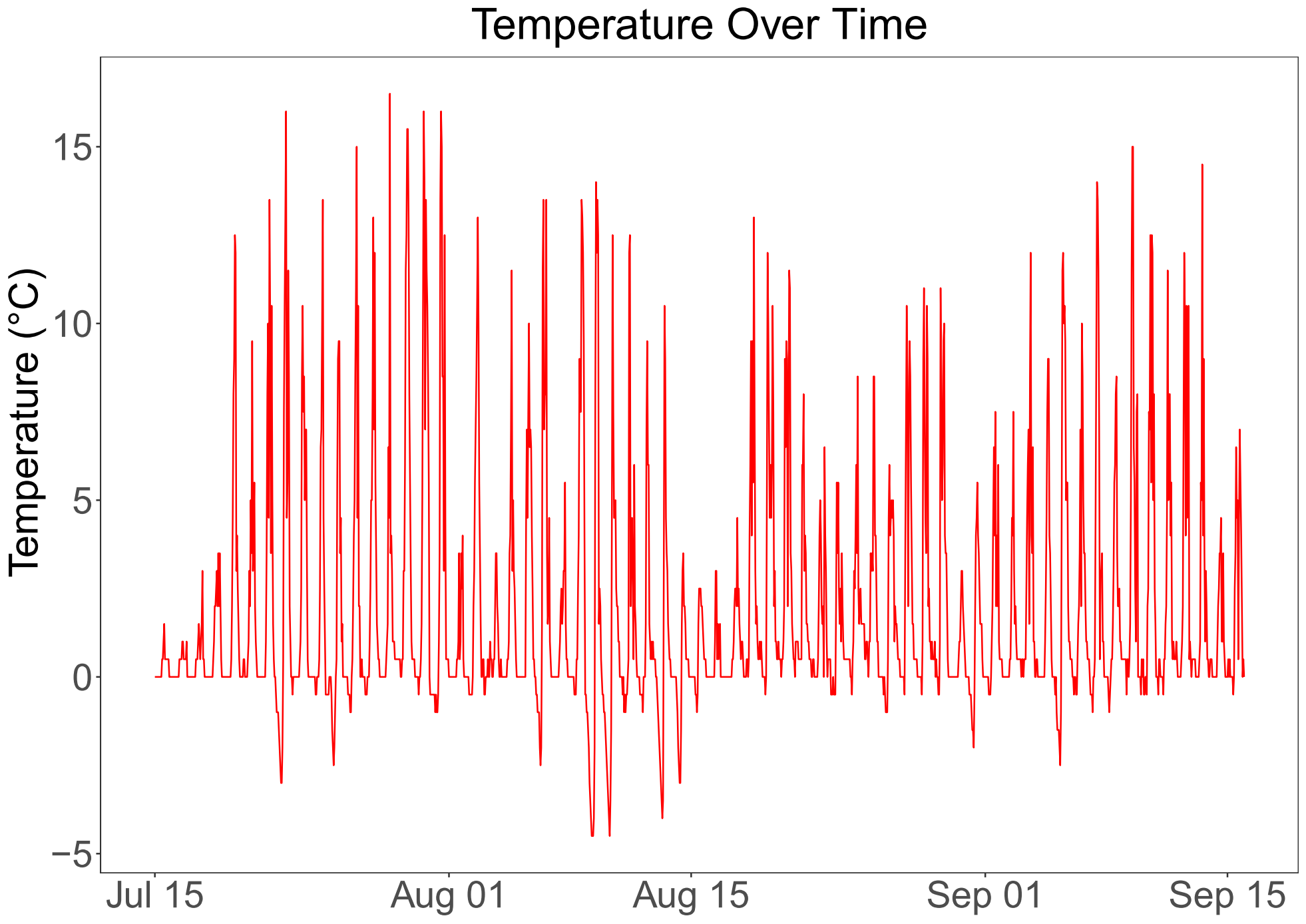


Figure 1: Temperature variations in the microenvironment of cryoconite holes from July 15 to September 15, 2019
